# Rural–Urban Disparities in Cancer Care—Analyzing Routinely Collected Patient‐Reported Outcomes. A Cross‐Sectional Study

**DOI:** 10.1002/cam4.70437

**Published:** 2025-04-15

**Authors:** Dominik J. Ose, Bayarmaa Mark, Krista Ocier, Emmanuel Adediran, Belinda Taylor, Kim Svoboda, Wallace Akerly, Brock O'Neil, Norah Lynn Henry, Mia Hashibe

**Affiliations:** ^1^ Department of Family and Preventive Medicine School of Medicine, University of Utah Salt Lake City Utah USA; ^2^ Faculty of Health and Healthcare Sciences Westsächsische Hochschule Zwickau Zwickau Saxony Germany; ^3^ Huntsman Cancer Institute, University of Utah Salt Lake City Utah USA; ^4^ Huntsman Cancer Registry University of Utah Hospital Salt Lake City Utah USA

## Abstract

**Objective:**

Rural–urban disparities in cancer care are well documented. However, research on rural–urban disparities regarding patient‐reported outcomes (PROs) is still developing. This study analyzed rural–urban disparities in patients with cancer with respect to anxiety, depression, fatigue, pain interference, and physical function.

**Methods:**

This study was conducted at the University of Utah Huntsman Cancer Institute. We integrated data from electronic health records, Cancer Registry, and PRO questionnaires. We assessed the association between rurality status (rural vs. urban) in patients with cancer and PRO scores using multiple linear regression models and t‐tests.

**Results:**

The cohort included 7271 patients. The mean age was 59.1 years at cancer diagnosis and 48.2% (*n* = 3505) were female. Across all cancer types, significant differences (Rural vs. Urban) were found for fatigue (53.6 vs. 54.1; *p* < 0.05) and physical function (45.5 vs. 45.1; *p* < 0.05). With respect to specific cancer types, there were differences in patients with *oral cavity and pharynx cancer* for depression (47.9 vs. 50.6; *p* < 0.01), fatigue (51.6 vs. 54.8; *p* < 0.05), pain interference (52.8 vs. 55.4; *p* < 0.05), and physical function (48.0 vs. 44.6; *p* < 0.01), *colorectal cancer* for fatigue (56.8 vs. 54.7; *p* < 0.05), pain interference (56.0 vs. 53.7; *p* < 0.05), and physical function (42.2 vs. 44.4; *p* < 0.05), *uterus cancer* for depression (47.5 vs. 50.5; *p* < 0.05) and fatigue (51.6 vs. 54.7; *p* < 0.05), and *lung cancer* for physical function (37.6 vs. 39.3; *p* < 0.05).

**Conclusions:**

Across all cancer types, as well as specific cancers, this study found mostly limited rural–urban differences regarding PROs. Except for colorectal and lung/bronchus cancer, patients living in rural areas reported similar or better PRO scores for all cancer types. Results support the hypothesis that improving access can help to level rural–urban disparities regarding cancer care outcomes, because all patients were treated in the same comprehensive cancer center, had similar access to care, and had similar PRO scores.

## 1 Background

In the United States (US), 84% of the country is considered rural, with approximately 57 million residents in these areas [1]. Compared to people living in urban areas, rural populations have lower educational attainment, encounter significantly more economic challenges (e.g., unemployment), and have higher percentages of uninsured and underinsured residents [2, 3, 4]. Additionally, rural residents tend to be older, more often engage in risky health behaviors (e.g., smoking), and are less engaged in preventive care [5, 6].

Although incidence of several cancers (e.g., breast cancer) are higher in urban areas [7, 8, 9], incidence of cancers highly associated with modifiable risks (e.g., lung cancer) and, overall, death rates are higher in rural populations [9, 10, 11]. Reasons for worse cancer outcomes in rural populations include challenges to accessing healthcare services (e.g., screening, prevention), lack of cancer care providers (e.g., oncologists, mental and behavioral health services), financial challenges, and transportation issues [12, 13, 14].

Patient‐reported outcomes (PROs) have evolved in recent years to become an integral part of clinical practice. Overall, PROs are valuable for evaluating quality of care and assessing subjective health status [15, 16, 17]. In patients with cancer, PROs help improve symptom management [18], identify psychosocial problems [19], facilitate patient‐centered care [20], support patient–provider communication [21, 22, 23] and improve clinical outcomes [24].

Previous studies on PRO disparities between rural and urban patients with cancer are inconclusive. In particular, previous research have assessed and compared subjective health status, for example, related to overall health‐related quality of life (HRQoL) [25], patient‐reported quality of care (e.g., care experience) [26], or mental health (e.g., anxiety).

Previous studies usually focused on one specific cancer, did not include clinical data and/or routinely collected PROs, or compared patients who were treated in a rural versus an urban healthcare setting. In contrast, this study analyses a large comprehensive sample of patients with a wide range of cancer types, all treated in the same comprehensive cancer center. The study integrates routine clinical data, cancer registry data, and routinely collected PROs. The aims of this study aims are to examine rural–urban disparities in PROs across cancer types with a focus on anxiety, depression, fatigue, pain interference, and physical function. We examined disparities for all cancer combined and for specific cancer types.

## 2 Methods

### 2.1 Design and Population

This retrospective cohort study was conducted at the University of Utah (UU) Huntsman Cancer Institute (HCI). The HCI is the only comprehensive cancer center in the Mountain West and serves the largest geographic region in the country (17% of U.S. continental land mass). Patients are drawn primarily from Utah, as well as from Nevada, Idaho, Wyoming, and Montana. The University of Utah Institutional Review Board (IRB #00126459) exempted the study.

### 2.2 Data Collection

Patients in this study were identified through the Huntsman Cancer Registry (HCR). The HCR contains cancer‐related information which were linked with routine clinical data available via the University of Utah Health Enterprise Data Warehouse. Data from both sources (Table S1) were collected between 2016 and 2019.

### 2.3 Inclusion/Exclusion Criteria

**FIGURE 1:**
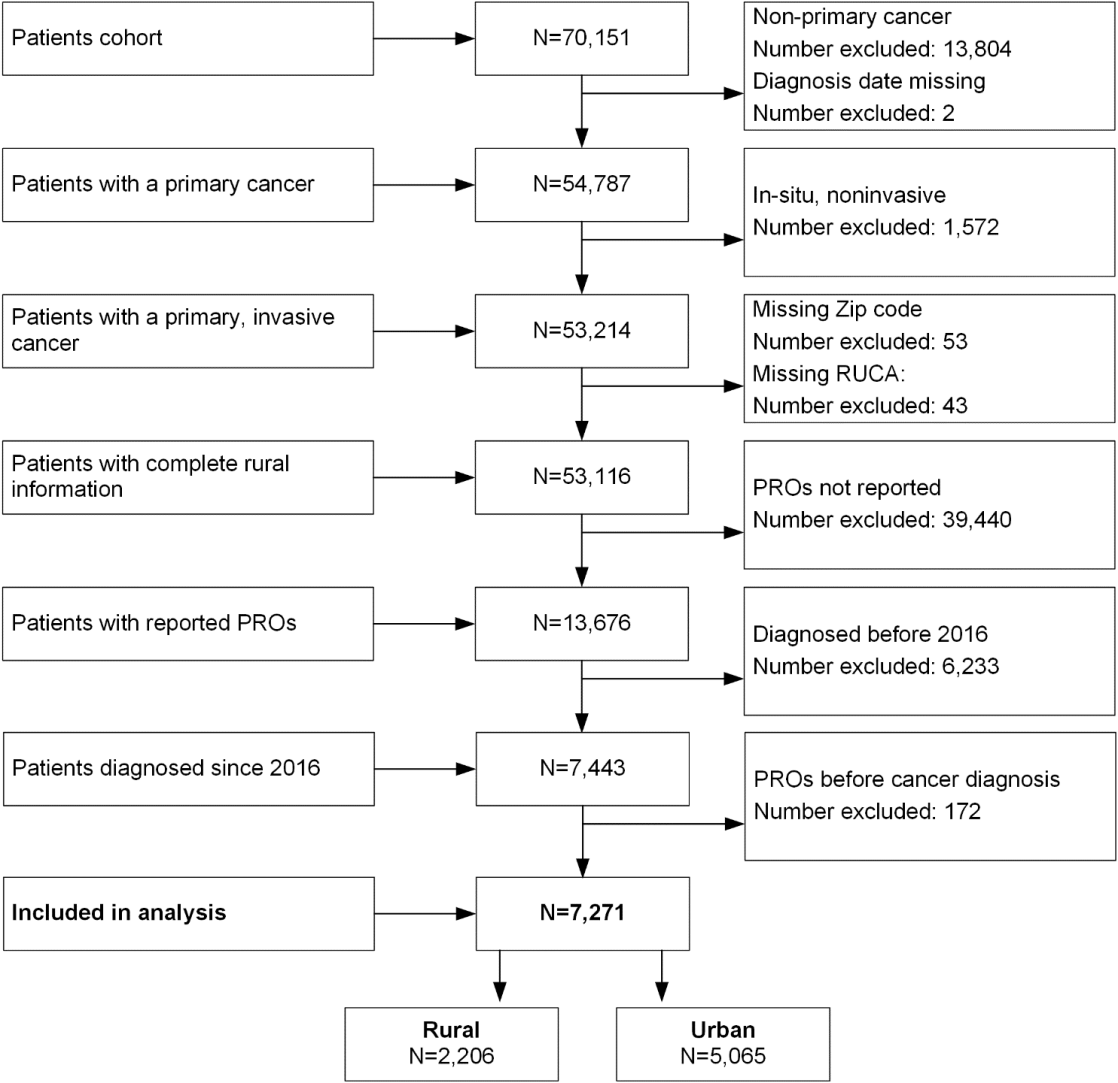
Flow chart of patient cohort.

Participant selection criteria included: age 18+ years, ICD‐O (International Classification of Diseases for Oncology) diagnosis, pathologically confirmed first primary invasive cancer, treated at the HCI between 2016 and 2019, and one or more reported PRO scores. Patients were excluded if the cancer was not primary or not invasive, the ZIP code was missing, PROs were not reported, patients were diagnosed before 2016, and PROs were only documented before cancer diagnosis (Figure 1).

### 2.4 Measures

#### 2.4.1 Patient‐Reported Outcomes (PRO)

In 2016, the University of Utah Health System (UU Health) adopted standardized PROs. The core assessment was based on PROMIS (Patient‐Reported Outcomes Measurement Information System) measures [27] and was performed by patients at least once a year and no more than once a week through tablet or email [28], via computerized adaptive testing (CAT). By using CAT, subsequent questions were based on answers to previous questions, which minimized the number of questions and the burden for the patient [29]. By design, PROMIS instruments reported outcomes utilizing standardized T‐scores (1–100) [30] and were calibrated to a mean *t*‐score of 50 with a standard deviation of 10 [31]. In this study, PROMIS instruments for anxiety, depression, fatigue, pain interference (lower score indicates better outcome), and physical functioning (higher score indicates better outcome) were included. All PROMIS measures have been validated for patients with cancer [32, 33, 34, 35, 36].

The PROMIS instruments for anxiety included fear, anxiousness, and arousal. Depression items consist of negative mood (e.g., sadness), reduction in positive effect, negative views of the self, negative social cognition, and challenges with decision‐making. Fatigue consisted of a constant sense of exhaustion which impacts the ability to complete daily activities of daily living (ADL) and social roles. Pain interference focuses on the impact of pain on daily living. Lastly, physical functioning was a self‐report of the ability to complete physical activities [27].

Reported as mean (SD) scores, the U.S. population‐based cancer values for PROMIS items included the following: Anxiety—49.2 (0.2); Depression—48.5 (0.2); Fatigue—52.2 (0.2); Pain—52.4 (0.2); and Physical functioning—44.8 (0.2) [33].

#### 2.4.2 Exposure

This study compared patients with cancer living in a rural patient with cancer living in urban areas (rurality status). Rurality status was based on Rural–Urban Commuting Area Codes (RUCA), based on patient ZIP codes [37].

#### 2.4.3 Covariates

Sex was binary coded as male or female rather than gender, which might exist on a spectrum and is most commonly used to indicate social or psychological differences between men, women, and other genders [38]. Other covariates included were *age at cancer diagnosis* (< 50 years, 50–64 years, 65–79 years, 80+ years); *race and ethnicity* (non‐Hispanic white, non‐Hispanic black; non‐Hispanic Asian; non‐Hispanic other; Hispanic), *BMI* (< 18.50: underweight, 18.50–24.99: normal weight, 25.00–29.99: overweight, 30.00+: obese), *marital status* (married, other), *state of residence* (Utah, Idaho, Wyoming, Nevada, Montana, other), *cancer stage* (stage I, stage II, stage III, stage IV), *cancer treatment* (Surgery, chemotherapy, radiation, hormone therapy, immunotherapy), and *primary cancer* (one primary cancer, multiple primary cancers).

### 2.5 Statistical Analysis

Clinicodemographic factors were assessed by rurality status (rural versus urban). For categorical variables, total counts and percentages are displayed, as well as means and standard deviations (SD) for continuous variables. We used Chi‐square tests for categorical variables and one‐way ANOVA for continuous variables to assess differences in clinicodemographic characteristics by rurality status. PRO scores were assessed using the means and SD. If PRO scores for an instrument (e.g., depression) were reported for more than one date after the cancer diagnosis, the average of all reported PRO scores for this instrument was used for analysis.

Multiple linear regression models were used to evaluate the association between rurality status and overall PRO scores, with t‐tests comparing the differences. PROs were adjusted for age at cancer diagnosis, sex, race/ethnicity, BMI, marital status, smoking status, cancer stage at diagnosis, and cancer type. These analyses were then stratified by several clinicodemographic moderators, including sex (Table S8), race and ethnicity (Table S9), and marital status (Table S10). Missing values were removed using list wise deletion. All statistical tests were two‐sided, and *p* < 0.05 were considered statistically significant.

## 3 Results

### 3.1 Clinical and Demographic Characteristics

A total of 7271 patients with cancer were included in the analyses; 2206 living in a rural area (30.3%) and 5065 living in an urban area (69.7%). The mean age was 59.1 years at cancer diagnosis, 48.2% of participants were female (*n* = 3505), 5.2% were Hispanic (*n* = 378), 88.1% were non‐Hispanic white (*n* = 6403), 36.8% were obese (*n* = 2677), and 69.9% were married (*n* = 5085). With respect to tumor sites, cancers of the breast (15.9%; *n* = 1157) and prostate (12.9%; *n* = 938) were the most common cancer diagnoses in this cohort. Regarding cancer severity and treatment, 31.2% (*n* = 1096) of all patients were diagnosed with stage III/IV cancer, 62.3% (*n* = 4533) had undergone surgery, and 46.2% (*n* = 3362) had undergone chemotherapy.

Compared to patients living in an urban area, patients living in a rural area were older (60.1 years vs. 58.7 years; *p* < 0.0001), more often male (54.5% vs. 50.6%; *p* < 0.01), more often non‐Hispanic white (90.1% vs. 86.8%; *p* > 0.001), were more often married (72.1% vs. 69.0%; *p* < 0.05), and more often living in Idaho, Wyoming, Nevada, and Montana (*p* < 0.0001). No statistical differences were found for cancer stage and cancer treatment (Table 1).

**TABLE 1:** Clinicodemographic characteristics (*n* = 7271)^a^.

Patients Characteristics	Population	Rural (*n* = 2206)	Urban (*n* = 5065)	*p* value
**Age** (**years**) **mean** (**SD**)^a^	59.1 (14.5)	60.1 (13.2)	58.7 (15.0)	**< 0.0001**
**Age categorized *n*** (**%**)^b^				
< 50 years	1654 (22.7%)	418 (18.9%)	1236 (24.4%)	**< 0.0001**
50–64 years	2696 (37.1%)	895 (40.6%)	1801 (35.6%)	
65–79 years	2562 (35.2%)	803 (36.4%)	1759 (34.7%)	
80+ years	359 (4.9%)	90 (4.1%)	269 (5.3%)	
**Sex *n*** (**%**)				
Female	3505 (48.2%)	1004 (45.5%)	2501 (49.4%)	**0.00257**
Male	3765 (51.8%)	1202 (54.5%)	2563 (50.6%)	
Unknown sex	1 (0.0)	—	1 (0.0)	
**Race and Ethnicity *n*** (**%**)				
Non‐Hispanic White	6403 (88.1%)	2009 (91.1%)	4394 (86.8%)	**< 0.0001**
Non‐Hispanic Black	48 (0.7%)	6 (0.3%)	42 (0.8%)	
Non‐Hispanic Asian	109 (1.5%)	10 (0.5%)	99 (2.0%)	
Non‐Hispanic Other^c^	183 (2.5%)	60 (2.7%)	123 (2.4%)	
Hispanic	378 (5.2%)	76 (3.4%)	302 (6.0%)	
Unknown	150 (2.1%)	45 (2.0%)	105 (2.1%)	
**BMI kg/m** ^**2**^ **mean** (**SD**)^d^	28.3 (6.72)	28.4 (6.34)	28.3 (6.88)	0.758
**BMI kg/m** ^**2**^ **category *n*** (**%**)^e^				
Underweight (< 18.5)	229 (3.1%)	68 (3.1%)	161 (3.2%)	0.348
Normal (18.5–24.99)	1916 (26.4%)	552 (25.0%)	1364 (26.9%)	
Overweight (25.0–29.99)	2409 (33.1%)	743 (33.7%)	1666 (32.9%)	
Obese (≥ 30)	2677 (36.8%)	834 (37.8%)	1843 (36.4%)	
Unknown	40 (0.6%)	9 (0.4%)	31 (0.6%)	
**Marital Status *n*** (**%**)				
Married	5085 (69.9%)	1590 (72.1%)	3495 (69.0%)	**0.0101**
Other	2006 (27.6%)	564 (25.6%)	1442 (28.5%)	
Unknown	180 (2.5%)	52 (2.4%)	128 (2.5%)	
**State of Residence *n*** (**%**)^f^				
Utah	5090 (70.0%)	779 (35.3%)	4311 (85.1%)	**< 0.0001**
Idaho	806 (11.1%)	345 (15.6%)	461 (9.1%)	
Wyoming	608 (8.4%)	597 (27.1%)	11 (0.2%)	
Nevada	401 (5.5%)	307 (13.9%)	94 (1.9%)	
Montana	134 (1.8%)	117 (5.3%)	17 (0.3%)	
Other	232 (3.2%)	61 (2.8%)	171 (3.4%)	
**Cancer Stage *n*** (**%**)				
Stage I	1847 (25.4%)	566 (25.7%)	1281 (25.3%)	0.218
Stage II	1515 (20.8%)	475 (21.5%)	1040 (20.5%)	
Stage III	628 (8.6%)	209 (9.5%)	419 (8.3%)	
Stage IV	1477 (20.3%)	435 (19.7%)	1042 (20.6%)	
Unknown^g^	1804 (24.8%)	521 (23.6%)	1283 (25.3%)	
**Cancer treatment *n*** (**%**)				
Surgery	4533 (62.3%)	1403 (63.6%)	3130 (61.8%)	0.588
Chemotherapy	3362 (46.2%)	994 (45.1%)	2368 (46.8%)	0.204
Radiation	2371 (32.6%)	727 (33.0%)	1644 (32.5%)	0.704
Hormone therapy	1660 (22.8%)	502 (22.8%)	1158 (22.9%)	0.959
Immunotherapy	1052 (14.5%)	301 (13.6%)	751 (14.8%)	0.203
**Primary cancer** (**%**)				
One primary cancer	6780 (93.2%)	2049 (92.9%)	4731 (93.4%)	0.444
Multiple primary cancer	491 (6.8%)	157 (7.1%)	334 (6.6%)	

*Note:* Bold values represent significance for readability.

Abbreviations: BMI = body mass index, *n* = number, SD = standard deviation.

^a^ Not all %s add up to 100 because of rounding decimal places.

^b^ At cancer diagnosis.

^c^ American Indian/Alaska Native, Hawaiian/Other Pacific Islander, Other, or Unknown.

^d^ Body Mass Index, at cancer diagnosis (90 days window before and after cancer diagnosis).

^e^ Body mass index category.

^f^ Determined from last known residence.

^g^ Brain and nervous system cancers are not routinely staged.

### 3.2 Responder vs. Non‐responder

Compared to patients who filled out the PRO questionnaire (responder), patients who did not fill out the PRO questionnaire (non‐responder) were more often male (53.9% vs. 51.8%; *p* < 0.05), more often Hispanic (7.1% vs. 5.2%; *p* < 0.0001), less often married (58.9% vs. 69.9%; *p* < 0.0001), less often diagnosed with stage III/IV cancer (20.8% vs. 28.9%; *p* < 0.0001), and less often received chemotherapy (*p* < 0.0001), radiation (*p* < 0.0001), hormone therapy (*p* < 0.0001), and immunotherapy (*p* < 0.0001) (Table S2).

### 3.3 PRO Scores Overall and by Cancer Type

**FIGURE 2:**
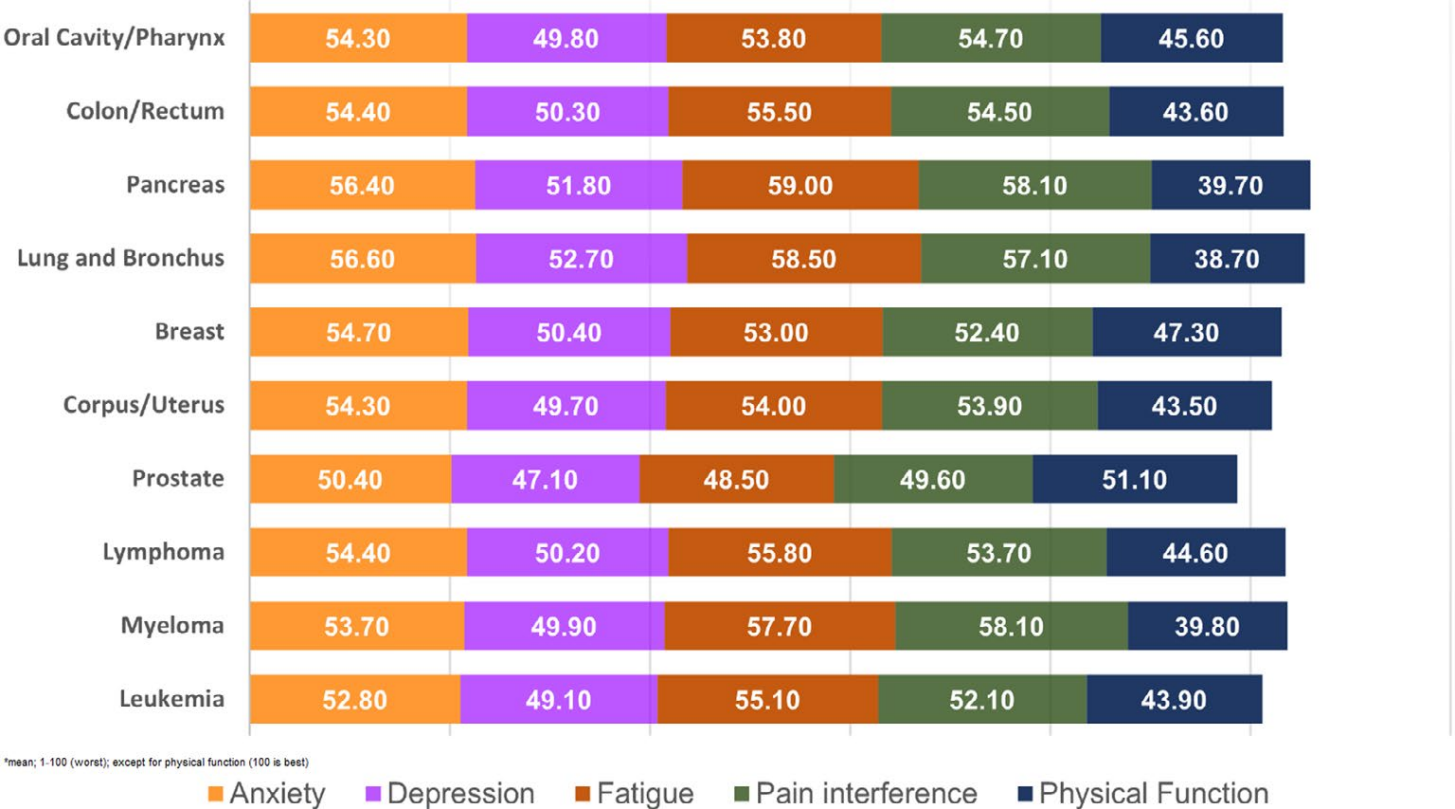
PRO scores by cancer type.

Across all cancer types, the mean scores were for anxiety 54.0 (SD 9.1), depression 49.9 (SD 8.8), for fatigue 54.0 (SD 10.5), pain interference 53.5 (SD 9.8), and physical function 45.2 (SD 10.7). However, noticeable differences in PRO scores existed by cancer types. PRO scores ranged for *anxiety* from 50.4 (prostate, SD 8.4) to 56.6 (lung and bronchus, SD 8.9), for *depression* from 47.1 (prostate, SD 8.1) to 52.7 (lung and bronchus, SD 9.0), for *fatigue* from 48.5 (prostate, SD 10.0) to 59.0 (pancreas, SD 9.7), for *pain interference* from 49.6 (prostate, SD 9.0) to 58.1 (pancreas, SD 9.8; Myeloma, SD 8.3), and for *physical function* from 38.7 (lung and bronchus, SD 8.9) to 51.1 (prostate, SD 9.7) (Table 2 and Figure 2).

**TABLE 2:** PRO scores by cancer type.

		Anxiety	Depression	Fatigue	Pain interference	Physical function
Cancer type	*N*	Mean (SD)	Mean (SD)	Mean (SD)	Mean (SD)	Mean (SD)
**All Cancer types**	7271	54.0 (9.1)	49.9 (8.8)	54.0 (10.5)	53.5 (9.8)	45.2 (10.7)
**Oral Cavity and Pharynx**	298	54.3 (9.1)	49.8 (9.1)	53.8 (10.4)	54.7 (9.6)	45.6 (10.3)
**Digestive System**	1228	55.2 (9.1)	51.0 (8.8)	57 (10.3)	55.8 (10.1)	42.0 (9.8)
Colon and Rectum	479	54.4 (9.1)	50.3 (8.8)	55.5 (10.4)	54.5 (10.3)	43.6 (9.8)
Pancreas	270	56.4 (9.6)	51.8 (8.8)	59.0 (9.7)	58.1 (9.8)	39.7 (8.8)
Other^a^	479	55.3 (8.9)	51.4 (8.9)	57.4 (10.3)	55.8 (9.9)	41.8 (10.0)
**Respiratory System**	590	56.5 (9.0)	52.5 (9.1)	58.2 (9.3)	57.0 (9.5)	39.1 (8.9)
Lung and Bronchus	510	56.6 (9.0)	52.7 (9.1)	58.5 (9.1)	57.1 (9.7)	38.7 (8.9)
Other^b^	80	55.6 (9.1)	51.1 (9.5)	56.4 (10.1)	56.4 (8.6)	41.5 (8.7)
**Skin** ^**c**^	596	51.4 (9.5)	47.4 (8.7)	49.3 (10.7)	50.1 (9.7)	50.7 (10.9)
Melanoma of the skin	489	51.6 (9.4)	47.5 (8.5)	49.4 (10.8)	50.4 (9.8)	50.6 (10.8)
Other^d^	107	50.6 (9.9)	46.9 (9.2)	48.4 (10.4)	48.4 (9.6)	51.5 (11.4)
**Breast**	1157	54.7 (8.9)	50.4 (8.3)	53.0 (9.8)	52.4 (9.0)	47.3 (9.9)
**Female Genital System**	408	54.9 (9.4)	50.5 (8.6)	55.2 (10.0)	54.7 (9.6)	43.2 (9.8)
Corpus and Uterus	187	54.3 (9.4)	49.7 (8.5)	54.0 (9.8)	53.9 (9.8)	43.5 (9.8)
Ovary	140	53.8 (8.7)	50.1 (8.3)	56.9 (8.7)	55.0 (9.2)	41.4 (8.9)
Other^e^	81	57.9 (10.0)	52.8 (9.1)	55.3 (11.9)	55.7 (10.1)	45.7 (11.1)
**Male Genital System**	1044	50.5 (8.5)	47.2 (8.3)	48.7 (10.1)	49.8 (9.1)	51.0 (9.8)
Prostate	938	50.4 (8.4)	47.1 (8.2)	48.5 (10.0)	49.6 (9.0)	51.1 (9.7)
Other^f^	106	51.8 (9.5)	48.2 (9.5)	50.6 (11.0)	51.0 (9.7)	50.2 (10.3)
**Urinary System**	436	54.3 (8.9)	49.8 (8.9)	54.5 (9.9)	54.7 (9.5)	44.0 (9.7)
Bladder	205	54.3 (8.6)	50.2 (8.6)	54.2 (9.0)	54.2 (9.33)	44.7 (9.8)
Kidney and Renal Pelvis	221	54.2 (9.2)	49.2 (9.1)	54.3 (10.7)	54.7 (9.5)	43.8 (9.5)
Other^g^	10	58.8 (6.0)	55.8 (6.5)	65.0 (7.8)	64.8 (6.3)	33.6 (7.4)
**Brain‐nervous system**	242	56.3 (9.1)	52.3 (8.8)	57.5 (9.2)	53.5 (9.7)	40.8 (10.5)
**Endocrine System**	182	54.1 (9.1)	49.9 (8.6)	52.9 (10.3)	50.3 (9.7)	50.2 (10.1)
Thyroid	159	53.9 (9.5)	49.7 (8.8)	52.8 (10.3)	49.8 (9.7)	50.9 (9.6)
Other^h^	23	55.8 (6.4)	51.4 (7.1)	53.4 (10.6)	53.5 (9.1)	45.7 (12.5)
**Lymphoma**	285	54.4 (8.4)	50.2 (8.1)	55.8 (9.5)	53.7 (9.8)	44.6 (9.9)
**Myeloma**	200	53.7 (8.7)	49.9 (8.7)	57.7 (9.1)	58.1 (8.3)	39.8 (9.2)
**Leukemia**	322	52.8 (9.0)	49.1 (9.1)	55.1 (10.1)	52.1 (9.5)	43.9 (10.0)
**Miscellaneous** ^**i**^	283	54.8 (8.5)	50.8 (8.5)	55.1 (10.7)	56.3 (9.6)	42.2 (10.5)

^a^ Esophagus, Stomach, Small Intestine, Liver, Intra Bile Duct, Anus, Gallbladder Other Biliary, Retroperitoneum, Peritoneum, Other Digestive Organs.

^b^ Nose, Larynx, Pleura, Trachea, Other.

^c^ Excluding Basal and Squamous.

^d^ Other Non‐Epithelial, Squamous Cell Carcinoma.

^e^ Cervix Uteri, Vagina, Vulva, Other Genital Organs; ^f^Testis, Penis, Other Genital Organs.

^g^ Ureter, Other Urinary Organs.

^h^ Other Endocrine System Organs.

^i^ Kaposi Sarcoma, Mesothelioma, Eye Orbit, Soft tissue, Bone Joints, other Miscellaneous.

### 3.4 PRO Scores Rural vs. Urban

Across all cancer types, there were only small differences between patients living in a rural area compared to an urban area. After adjusting for sex, age of cancer diagnosis, marital status, cancer stage, cancer type, BMI, and race/ethnicity, significant differences for rural versus urban existed for fatigue (53.6 vs. 54.1; β ± SE: −0.516 ± 0.26; *p* < 0.05) and physical function (45.5 vs. 45.1; β ± SE: 0.0509 ± 0.25; *p* < 0.05) (Table 3).

**TABLE 3:** PRO scores rural vs. urban (*n* = 7271).

PRO Scores	Population		Rural (*n* = 2206)		Urban (*n* = 5065)		*p* ^a^	*p* ^b^	β ± SE^b^
	*N*		*N*	Mean (SD)	*N*	Mean (SD)			
Anxiety	7145	54.0 (9.1)	2171	53.8 (9.1)	4974	54.0 (9.2)	0.407	0.5795	−0.129 ± 0.23
Depression	7127	49.9 (8.8)	2162	49.7 (8.8)	4965	50.0 (8.8)	0.142	0.1364	−0.336 ± 0.23
Fatigue	7161	54.0 (10.5)	2174	53.6 (10.7)	4987	54.1 (10.4)	0.0607	**0.0459**	−0.516 ± 0.26
Pain interference	7189	53.5 (9.8)	2177	53.4 (10.0)	5012	53.5 (9.8)	0.592	0.4127	−0.202 ± 0.25
Physical Function	7217	45.2 (10.7)	2187	45.5 (10.8)	5030	45.1 (10.6)	0.13	**0.0385**	0.509 ± 0.25

^a^ Unadjusted: comparing only means.

^b^ Adjusted for sex, age of cancer diagnosis, marital status, cancer stage, cancer type, BMI, and race/ethnicity.

**FIGURE 3:**
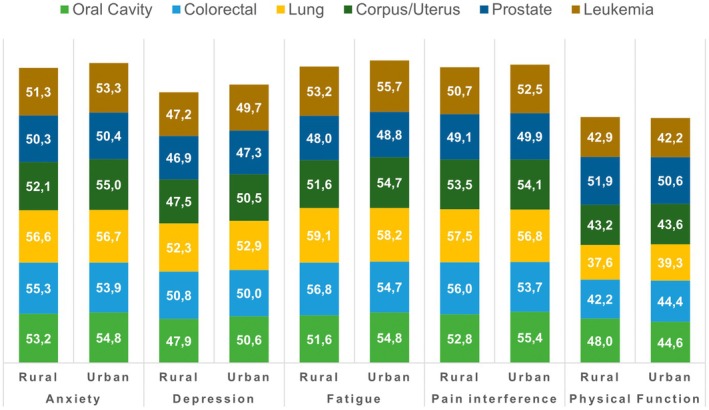
PRO scores by cancer type and rurality (rural vs. urban).

However, differences in PRO scores between rural and urban patients strongly varied with respect to the specific tumor site and the specific PRO measure. Significant differences (adjusted) were observed, for example, in patients with *oral cavity cancer* for depression (47.9 vs. 50.6; *p* < 0.01), *fatigue* (51.6 vs. 54.8; *p* < 0.05), pain interference (52.8 vs. 55.4; *p* < 0.05), and physical function (48.0 vs. 44.6; *p* < 0.01), *colorectal cancer* for fatigue (56.8 vs. 54.7; *p* < 0.05), pain interference (56.0 vs. 53.7; *p* < 0.05), and physical function (42.2 vs. 44.4; *p* < 0.05), *uterus cancer* for depression (47.5 vs. 50.5; *p* < 0.05), fatigue (51.6 vs. 54.7; *p* < 0.05), or *lung cancer* for physical function (37.6 vs. 39.3; *p* < 0.05) (Table 4 and Figure 3).

**TABLE 4:** PRO scores by rurality status for selected tumor sites^a^.

Cancer type	*N*	Anxiety		Depression		Fatigue		Pain interference		Physical function	
		Rural	Urban	Rural	Urban	Rural	Urban	Rural	Urban	Rural	Urban
		Mean (SD)	Mean (SD)	Mean (SD)	Mean (SD)	Mean (SD)	Mean (SD)	Mean (SD)	Mean (SD)	Mean (SD)	Mean (SD)
Oral Cavity^1^	298	53.2 (8.0)	54.8 (9.1)	47.9 (7.8)	50.6 (9.5)^#^*	51.6 (11.0)	54.8 (10.1) ^#^*	52.8 (10.6)	55.4 (9.2) ^#^*	48.0 (10.0)	44.6 (10.33)^##^**
Colorectal	479	55.3 (9.3)	53.9 (9.0)	50.8 (8.9)	50.0 (8.7)	56.8 (10.0)	54.7 (10.6) ^#^*	56.0 (10.3)	53.7 (10.2) ^#^*	42.2 (9.2)	44.4 (10.6)^#^*
Lung^2^	510	56.6 (9.1)	56.7 (8.9)	52.3 (9.0)	52.9 (9.0)	59.1 (8.7)	58.2 (9.4)	57.5 (9.7)	56.8 (9.4)	37.6 (8.1)	39.3 (9.6)^#^*
Corpus/Uterus	187	52.1 (8.9)	55.0 (9.4)	47.5 (7.7)	50.5 (8.6)^#^	51.6 (9.5)	54.7 (9.8)^#^	53.5 (9.1)	54.1 (10.0)	43.2 (9.7)	43.6 (9.8)
Prostate	938	50.3 (8.6)	50.4 (8.2)	46.9 (8.5)	47.3 (8.0)	48.0 (10.3)	48.8 (9.8)	49.1 (8.9)	49.9 (9.1)	51.9 (9.3)	50.6 (10.0)^#^
Leukemia	322	51.3 (8.5)	53.3 (9.1)	47.2 (9.4)	49.7 (9.0)^#^*	53.2 (10.0)	55.7 (10.1)*	50.7 (9.6)	52.5 (9.5)	42.9 (10.8)	42.2 (9.6)

*Note:* ^1^and Pharynx; ^2^and Bronchus; *p* unadjusted: ^#^ *p* < 0.05; ^##^ *p* < 0.01; ^###^ *p* < 0.001; *p* adjusted: *p < 0.05; **p < 0.01; ***p < 0.001.

^a^ A complete overview for all tumor sites can be found in the online‐appendix.

### 3.5 PRO Scores by Clinicodemographics

Stratified by clinicodemographics, significant differences comparing rural vs. urban patients were observed in non‐Hispanic white patients for *depression* (49.5 vs. 50.0; β ± SE: −0.480 ± 0.23; *p* < 0.05), *fatigue* (53.5 vs. 54.3; β ± SE: −0.732 ± 0.27; *p* < 0.01), and for *physical function* (45.8 vs. 45.2; β ± SE: 0.662 ± 0.26; *p* < 0.01) (Table S8), as well as in married patients for *depression* (48.7 vs. 49.0; β ± SE: −0.647 ± 0.26; *p* < 0.05), *fatigue* (52.5 vs. 53.1; β ± SE: −0.790 ± 0.30; *p* < 0.01), *pain interference* (52.9 vs. 53.1; β ± SE: −0.642 ± 0.29; *p* < 0.05), and for *physical function* (46.5 vs. 46.1; β ± SE: 0.857 ± 0.29; *p* < 0.01) (Table S9). No differences were observed by gender (Table S10).

## 4 Discussion

This study aimed to analyze rural–urban disparities in patients with cancer regarding anxiety, depression, fatigue, pain interference, and physical function across all cancer types, as well as specific cancer types. The analysis revealed three main findings. **First:** Across all cancer types, no significant rural–urban differences for anxiety, depression, and pain interference, and only small, not clinically relevant, statistically significant differences for fatigue and physical function were observed. **Second:** For the majority of analyzed cancer types, no differences we observed for any of the included PROs. **Third:** For some cancer types, rural patients had better PRO scores regarding, for example, depression (e.g., uterus cancer, leukemia), fatigue (e.g., oral cavity and pharynx cancer), or physical activity (e.g., prostate cancer), as well as worse PRO scores regarding fatigue (colorectal cancer), pain interference (colorectal cancer), and physical activity (colorectal cancer, lung cancer). However, not all of those differences are clinically meaningful.

Several previous studies reported that patients with cancer living in rural areas were more often affected by anxiety or other mental health issues [39, 40, 41]. For example, the study of Burris and Andrykowski reported that, compared to urban cancer survivors, rural cancer survivors had shown worse mental health functioning, with more symptoms of anxiety and depression, as well as more emotional problems than non‐rural cancer survivors [39]. In contrast, we did not observe any difference in anxiety scores, neither across all cancer types nor with respect to any specific cancer type.

Also in contrast to previous studies [42, 43, 44, 45], we did not observe any rural–urban disparities regarding depression, fatigue, and physical function, except for colorectal cancer (fatigue, pain interference, physical function) and lung and bronchus cancer (physical function). However, for the first time, to the best of our knowledge, we observed better patient‐reported outcomes for patients living in a rural area for specific some cancer types (oral cavity/pharynx, corpus/uterus, leukemia, prostate).

Importantly, we acknowledge the distinction between statistical differences and what is considered minimally important differences (MID) or clinically meaningful differences. A prior study on interpreting PROs in cancer cohorts recommended the following t‐score ranges for MID: fatigue (2.5–5.0), pain interference (4.0–6.0), physical functioning (4.0–6.0), anxiety (3.0–4.5), and depression (3.0–4.5) [46]. Based on these criteria, there were no important differences, overall, in PROs between rural and urban cancer patients in our study. However, by cancer type, there were meaningful differences for oral cavity (fatigue and physical function), corpus/uterus (depression and fatigue), and leukemia (fatigue).

Generally speaking, research on rural–urban disparities regarding PROs in patients with cancer is still developing and inconclusive. Other than the PROs focused on herein (e.g., mental health outcomes, pain interference, physical function), several studies addressed rural–urban disparities with respect to HRQoL. Many of those studies were focused on breast [25, 47, 48] and prostate cancer [40, 41, 49, 50]. While some studies reported that patients with cancer living in rural areas have worse HRQoL [25, 48, 51, 52], other studies reported no differences [47, 50].

Often discussed reasons for rural–urban disparities regarding PROs are challenges in accessing appropriate healthcare services include lack of cancer care providers and transportation issues [49, 53, 54]. In contrast to previous studies that compared patients treated in rural versus urban health centers, in our study, all patients received treatment at the same comprehensive cancer center. Our results support the hypothesis that improved access to cancer care can mitigate rural–urban disparities in outcomes. Similarly, Unger et al. demonstrated that rural and urban patients with comparable access to care through clinical research participation had similar disease outcomes [55].

## 5 Strengths and Limitations

This study has several strengths. This analysis is based on a large real‐world cancer patient population from the Western US (Utah, Idaho, Wyoming, Nevada, Montana). Our population was restricted to patients diagnosed with pathologically confirmed first primary invasive cancer. Through the innovative linkage of the Huntsman Cancer Registry and the University of Utah Health Enterprise Data Warehouse (EDW), a wealth of clinical and study‐related data was available for this analysis.

However, this study also has limitations [1]. Part of this analysis is based on routine clinical data. Limitations of routine clinical data include, missing, inaccurate, and incomplete data, and restrictions of fixed data fields. To overcome those limitations, we thoroughly checked and cleaned the data set, used mostly cancer registry data, and minimized the use of routine clinical patient‐level data [2]. Because the PROs used for this analysis are routinely collected, the number of available PROs and the time points where the PROs were filled out varied. To address variation, the average of each reported PRO (anxiety, depression, fatigue, pain interference, and physical function) was used for analysis [3]. PROs were only available in English, which may also explain the higher proportion of Hispanics in the non‐responder group [4]. There could be a selection bias. For example, in order for a rural patient from Montana to travel such a distance for care suggests a minimum level of socioeconomic resources and functional capacity. Such resources and capacity may directly contribute to better outcomes among rural patients [5]. We did not specifically analyze the number of repeated PRO assessments between urban and rural patients, which could potentially impact our findings. Urban cancer patients may have received more follow‐up care at the Huntsman Cancer Institute, and rural cancer patients may have accessed more follow‐up care locally, resulting in fewer longitudinal PRO scores post‐treatment [6]. Generalizability is also limited since patients in our study received care from a single comprehensive care center. Our results may differ from studies conducted at federal, private, or other academic healthcare centers.

## 6 Conclusion

Across all cancer types, as well as specific cancers, this study found mostly limited rural–urban differences regarding PROs. Except for colorectal and lung/bronchus cancer, patients living in rural areas reported similar or better PRO scores for all cancer types. Results support the hypothesis that improving access can help to level rural–urban disparities regarding cancer care outcomes, because all patients included in this study received treatment at the same comprehensive cancer center, had similar access to care, and had similar PRO scores.

## Author Contributions

**Dominik J. Ose:** conceptualization (lead), data curation (lead), formal analysis (equal), funding acquisition (lead), investigation (lead), methodology (lead), project administration (equal), resources (lead), software (equal), supervision (lead), validation (lead), visualization (lead), writing – original draft (lead), writing – review and editing (lead). **Bayarmaa Mark:** formal analysis (supporting), investigation (supporting), methodology (supporting), software (supporting), validation (supporting), visualization (supporting), writing – original draft (supporting), writing – review and editing (supporting). **Krista Ocier:** formal analysis (lead), methodology (supporting), validation (supporting), visualization (supporting). **Emmanuel Adediran:** formal analysis (supporting), investigation (supporting), methodology (supporting), project administration (lead), supervision (supporting), writing – review and editing (supporting). **Belinda Taylor:** data curation (lead). **Kim Svoboda:** data curation (lead). **Wallace Akerly:** methodology (supporting), validation (supporting), writing – original draft (supporting), writing – review and editing (supporting). **Brock O'Neil:** investigation (supporting), methodology (supporting), validation (supporting), writing – original draft (supporting), writing – review and editing (supporting). **Norah Lynn Henry:** investigation (supporting), methodology (supporting), validation (supporting), writing – original draft (supporting), writing – review and editing (supporting). **Mia Hashibe:** conceptualization (equal), data curation (equal), formal analysis (supporting), investigation (supporting), methodology (supporting), supervision (lead), validation (supporting), visualization (supporting), writing – original draft (supporting), writing – review and editing (supporting).

## Conflicts of Interest

The authors declare no conflicts of interest.

## Supporting information


Data S1.

## Data Availability

The data that support the findings of this study are available from the corresponding author upon request.
