# Supplementary material for: Rural–Urban Disparities in Cancer Care—Analyzing Routinely Collected Patient‐Reported Outcomes. A Cross‐Sectional Study
Source: Cancer Med. 2025 Apr 15;14(8):e70437. doi: 10.1002/cam4.70437 (PMC11997707; doi:10.1002/cam4.70437)
Supplement: Supplementary file 1 — Data S1. [file CAM4-14-e70437-s001.docx]

**Supplementary Online Content**

**eTable 1:** Data description

**eTable 2:** Non-Responder analysis

**eTable 3:** Anxiety by cancer type and rural vs urban

**eTable 4:** Depression by cancer type and rural vs urban

**eTable 5:** Fatigue by cancer type and rural vs urban

**eTable 6:** Pain Inference by cancer type and rural vs urban

**eTable 7:** Physical Function by cancer type and rural vs urban

**eTable 8:** PRO scores by race/ethnicity and rurality status

**eTable 9**: PRO scores by marital status and rurality status

**eTable 10**: PRO scores by gender and rurality status

**eTable 1:** Data description

| **Type and name** | | **Source** | **Description** | **Coding (example)** |
| --- | --- | --- | --- | --- |
| Demographics | | | | |
|  | Date of birth (DOB) | EDW | Patient-reported | 04/12/1978 |
|  | Age (at cancer diagnosis) | EDW | Calculated, based on DOB and Date of cancer diagnosis | 59 |
|  | Age categorized (at cancer diagnosis) | EDW | Calculated, based on DOB and Date of cancer diagnosis | <50 years; 50-64 years; 65-79 years; 80+ years |
|  | Sex | EDW | Patient-reported | Male; Female |
|  | BMI (mean) | EDW | Calculated, based on  clinical measures | 25.3 |
|  | BMI groups | EDW | Calculated, based on  clinical measures | Underweight <18.5; Healthy weight 18.5 to 24.9; Overweight 25.0 to 29.9; Obese 30+ |
|  | Marital status categorized | EDW | Calculated, based on more detailed marital information | Married; Other; Unknown |
|  | Race and ethnicity | EDW | Patient-reported | Non-Hispanic white; Non-Hispanic black; Non-Hispanic Asian; Non-Hispanic other; Hispanic |
|  | State of residence | EDW | Patient-reported | Utah; Idaho; Wyoming; Nevada; other |
|  | Population | EDW | Calculated, based on  RUCA codes using ZIP | Urban; Rural |
| Patient reported Outcomes | | | | |
|  | Date | EDW | Date documented each time PROs were collected | 02/03/2017 |
|  | PRO method | EDW | Method for collecting PROS | Email; Tablet at visit |
|  | Anxiety score | EDW | PROMIS score for anxiety  ranging 0-100 (100=worst) | 56 |
|  | Depression score | EDW | PROMIS score for depression ranging 0-100 (100=worst) | 49 |
|  | Fatigue score | EDW | PROMIS score for fatigue ranging 0-100 (100=worst) | 34 |
|  | Pain interference score | EDW | PROMIS score for pain interference ranging 0-100 (100=worst) | 45 |
|  | Score for physical function |  | PROMIS score for physical function ranging 0-100 (100=best) | 59 |
| Cancer characteristics | | | | |
|  | Cancer diagnosis | HCR | First cancer diagnosis documented in HCR | 01/03/2017 |
|  | ICD-O diagnosis*** | HCR | Documented ICD-O Histo-code and Site-code | 8120/3; C67.9 |
|  | Stage | HCR | Documented “Best Dominant Stage” | 1a |
|  | Age at diagnosis | HCR | Calculated, based on DOB and cancer diagnosis Date | 48 years |
|  | Treatment | HCR | Abstracted form HCR trough certified tumor registrars | Surgery, chemotherapy, radiation, hormone therapy, immunotherapy |
|  | Sequence Number** | HCR | Sequence of tumor | 01 |

* To confirm diagnosis, 2+ laboratory test results more than two days apart were required if no other indication of diagnosis was given (ICD code, insulin prescription); ** Restricted to 0 or 1 (primary cancers); *** Restricted /3 (invasive cancer, primary site): HCR: Huntsman Cancer Institute; EDW: Enterprise Data Warehouse

**eTable 2:** Non-Responder analysis (n=11,603)^1^

| **Patients Characteristics** | **Population** | **Responder** (n=7,271) | **Non-Responder** (n=4,332) | **p-value** |
| --- | --- | --- | --- | --- |
| **Age (years) mean (SD)**^1^ | 59.2 (16.1) | 59.1 (14.5) | 59.2 (18.5) | 0.665 |
| **Age categorized n (%)**^2^ |  |  |  | **<.0001** |
| <50 years | 2725 (23.5%) | 1654 (22.7%) | 1071 (24.7%) |  |
| 50 to 64 years | 3994 (34.4%) | 2696 (37.1%) | 1298 (30.0%) |  |
| 65 to 79 years | 4072 (35.1%) | 2562 (35.2%) | 1510 (34.9%) |  |
| 80+ years | 812 (7.0%) | 359 (4.9%) | 453 (10.5%) |  |
| **Sex n (%)** |  |  |  | **0.0315** |
| Female | 5503 (47.4%) | 3505 (48.2%) | 1998 (46.1%) |  |
| Male | 6098 (52.6%) | 3765 (51.8%) | 2333 (53.9%) |  |
| Unknown sex | 2 (0.0%) | 1 (0.0%) | 1 (0.0%) |  |
| **Race and Ethnicity n (%)** |  |  |  | **<.0001** |
| Non-Hispanic White | 9948 (85.7%) | 6403 (88.1%) | 3545 (81.8%) |  |
| Non-Hispanic Black | 87 (0.7%) | 48 (0.7%) | 39 (0.9%) |  |
| Non-Hispanic Asian | 183 (1.6%) | 109 (1.5%) | 74 (1.7%) |  |
| Non-Hispanic Other^3^ | 317 (2.7%) | 183 (2.5%) | 134 (3.1%) |  |
| Hispanic | 684 (5.9%) | 378 (5.2%) | 306 (7.1%) |  |
| Unknown | 384 (3.3%) | 150 (2.1%) | 234 (5.4%) |  |
| **BMI kg/m^2^ mean (SD)**^4^ | 28.4 (7.76) | 28.3 (6.72) | 28.5 (9.35) | 0.315 |
| **BMI kg/m^2^ category n (%)**^5^ |  |  |  | **<.0001** |
| Underweight (<18.5) | 435 (3.7%) | 229 (3.1%) | 206 (4.8%) |  |
| Normal (18.5 - 24.99) | 2977 (25.7%) | 1916 (26.4%) | 1061 (24.5%) |  |
| Overweight (25.0 - 29.99) | 3644 (31.4%) | 2409 (33.1%) | 1235 (28.5%) |  |
| Obese (>=30) | 4206 (36.2%) | 2677 (36.8%) | 1529 (35.3%) |  |
| Unknown | 341 (2.9%) | 40 (0.6%) | 301 (6.9%) |  |
| **Marital Status n (%)** |  |  |  | **<.0001** |
| Married | 7638 (65.8%) | 5085 (69.9%) | 2553 (58.9%) |  |
| Other | 3569 (30.8%) | 2006 (27.6%) | 1563 (36.1%) |  |
| Unknown marital | 396 (3.4%) | 180 (2.5%) | 216 (5.0%) |  |
| **State of Residence n (%)**^6^ |  |  |  | **0.0114** |
| Utah | 8154 (70.3%) | 5090 (70.0%) | 3064 (70.7%) |  |
| Idaho | 1277 (11.0%) | 806 (11.1%) | 471 (10.9%) |  |
| Wyoming | 905 (7.8%) | 608 (8.4%) | 297 (6.9%) |  |
| Nevada | 646 (5.6%) | 401 (5.5%) | 245 (5.7%) |  |
| Montana | 244 (2.1%) | 134 (1.8%) | 110 (2.5%) |  |
| Other | 377 (3.2%) | 232 (3.2%) | 145 (3.3%) |  |
| **Cancer Stage n (%)** |  |  |  | **<.0001** |
| Stage I | 3141 (27.1%) | 1847 (25.4%) | 1294 (29.9%) |  |
| Stage II | 2119 (18.3%) | 1515 (20.8%) | 604 (13.9%) |  |
| Stage III | 896 (7.7%) | 628 (8.6%) | 268 (6.2%) |  |
| Stage IV | 2115 (18.2%) | 1477 (20.3%) | 638 (14.7%) |  |
| Unknown^7^ | 3332 (28.7%) | 1804 (24.8%) | 1528 (35.3%) |  |
| **Cancer treatment n (%)** |  |  |  |  |
| Surgery | 7005 (60.4%) | 4533 (62.3%) | 2472 (57.1%) | **0.0005** |
| Chemotherapy | 4436 (38.2%) | 3362 (46.2%) | 1074 (24.8%) | **<.0001** |
| Radiation | 3281 (28.3%) | 2371 (32.6%) | 910 (21.0%) | **<.0001** |
| Hormone therapy | 2258 (19.5%) | 1660 (22.8%) | 598 (13.8%) | **<.0001** |
| Immunotherapy | 1358 (11.7%) | 1052 (14.5%) | 306 (7.1%) | **<.0001** |
| **Primary Cancer (%)** |  |  |  | **<.0001** |
| One primary cancer | 10949 (94.4%) | 6780 (93.2%) | 4169 (96.2%) |  |
| Multiple primary cancer | 654 (5.6%) | 491 (6.8%) | 163 (3.8%) |  |

^1^ Not all %s add up to 100 because of rounding decimal places; ^2^ At cancer diagnosis; ^3^ American Indian/Alaska Native, Hawaiian/Other Pacific Islander, Other, or Unknown; ^4^ Body Mass Index, at cancer diagnosis (90 days window before and after cancer diagnosis); ^5^ Body mass index category; ^6^ Determined from last known residence; ^7^ Brain and nervous system cancers are not routinely staged; n=number; SD=standard deviation; BMI=body mass index

**eTable 3:** Anxiety by cancer type and rural vs urban (n=7,271)

|  | **Rural** (n=2,206) | | **Urban** (n=5,065) | |  |  |
| --- | --- | --- | --- | --- | --- | --- |
| **Cancer Type** | N | Mean (SD) | N | Mean (SD) | **P**^1^ | **P**^2^ |
| **Oral Cavity and Pharynx** | 87 | 53.2 (7.98) | 206 | 54.8 (9.46) | 0.13959 | 0.14626 |
| **Digestive System** | 381 | 55.8 (9.13) | 824 | 54.9 (9.14) | 0.11586 | **0.05765** |
| Colon and Rectum | 169 | 55.3 (9.34) | 300 | 53.9 (8.98) | 0.11739 | 0.09105 |
| Pancreas | 74 | 56.6 (10.20) | 193 | 56.3 (9.33) | 0.81845 | 0.59884 |
| Other^a^ | 138 | 56.0 (8.27) | 331 | 55.0 (9.07) | 0.25514 | 0.16353 |
| **Respiratory System** | 202 | 56.2 (9.01) | 382 | 56.6 (8.93) | 0.59900 | 0.28989 |
| Lung and Bronchus | 174 | 56.6 (9.05) | 331 | 56.7 (8.87) | 0.92743 | 0.56996 |
| Other^b^ | 28 | 54.0 (8.53) | 51 | 56.5 (9.38) | 0.23748 | 0.49655 |
| **Skin^c^** | 150 | 51.3 (9.12) | 441 | 51.4 (9.64) | 0.86042 | 0.75116 |
| Melanoma of the skin | 130 | 51.5 (9.45) | 354 | 51.6 (9.42) | 0.93818 | 0.80857 |
| Other^d^ | 20 | 49.8 (6.60) | 87 | 50.8 (10.52) | 0.58570 | 0.99781 |
| **Breast** | 350 | 54.6 (8.80) | 787 | 54.8 (8.93) | 0.79009 | 0.43501 |
| **Female Genital System** | 100 | 54.3 (9.14) | 298 | 55.1 (9.45) | 0.46381 | 0.38225 |
| Corpus and Uterus | 45 | 52.1 (8.85) | 135 | 55.0 (9.43) | 0.06677 | 0.19522 |
| Ovary | 33 | 53.6 (9.11) | 104 | 53.9 (8.62) | 0.83599 | 0.70070 |
| Other^e^ | 22 | 59.8 (7.79) | 59 | 57.2 (10.64) | 0.23829 | 0.58505 |
| **Male Genital System** | 366 | 50.3 (8.82) | 662 | 50.7 (8.31) | 0.47838 | 0.72107 |
| Prostate | 339 | 50.3 (8.64) | 585 | 50.4 (8.21) | 0.90283 | 0.91782 |
| Other^f^ | 27 | 49.3 (10.98) | 77 | 52.6 (8.83) | 0.17226 | 0.31596 |
| **Urinary System** | 122 | 54.1 (8.90) | 307 | 54.4 (8.88) | 0.71270 | 0.48539 |
| Bladder | 53 | 54.0 (8.52) | 149 | 54.4 (8.66) | 0.77870 | 0.80929 |
| Kidney and Renal Pelvis | 67 | 54.0 (9.28) | 150 | 54.2 (9.21) | 0.88009 | 0.72244 |
| Other^g^ | 2 | 58.1 (9.72) | 8 | 59.0 (5.69) | 0.92213 | - |
| **Brain-nervous system** | 61 | 56.2 (8.41) | 177 | 56.3 (9.40) | 0.92416 | 0.82189 |
| **Endocrine System** | 54 | 54.6 (8.93) | 121 | 53.9 (9.27) | 0.65144 | 0.17583 |
| Thyroid | 48 | 54.3 (9.36) | 104 | 53.7 (9.58) | 0.67384 | 0.23930 |
| Other^h^ | 6 | 56.5 (4.24) | 17 | 55.5 (7.04) | 0.69699 | 0.93239 |
| **Lymphoma** | 78 | 54.7 (7.7) | 199 | 54.3 (8.7) | 0.71819 | 0.71075 |
| **Myeloma** | 54 | 54.7 (9.0) | 145 | 53.4 (8.57) | 0.36538 | 0.23037 |
| **Leukemia** | 77 | 51.3 (8.47) | 235 | 53.3 (9.08) | 0.08694 | 0.08504 |
| **Miscellaneous^i^** | 89 | 54.1 (9.0) | 190 | 55.1 (8.25) | 0.35370 | 0.35496 |

^1^Unadjusted t-test p-value; ^2^Adjusted for sex, age of cancer diagnosis, marital status, smoking status, cancer stage, BMI, and race/ethnicity; ^a^Esophagus, Stomach, Small Intestine, Liver, Intra Bile Duct, Anus, Gallbladder Other Biliary, Retroperitoneum, Peritoneum, Other Digestive Organs; ^b^Nose, Larynx, Pleura, Trachea, Other; ^c^excluding Basal and Squamous; ^d^Other Non-Epithelial, Squamous Cell Carcinoma; ^e^Cervix Uteri, Vagina, Vulva, Other Genital Organs; ^f^Testis, Penis, Other Genital Organs; ^g^Ureter, Other Urinary Organs; ^h^Other Endocrine System Organs; ^i^Kaposi Sarcoma, Mesothelioma, Eye Orbit, Soft tissue, Bone Joints, other Miscellaneous

**eTable 4:** Depression by cancer type and rural vs urban (n=7,271)

|  | **Rural** (n=2,206) | | **Urban** (n=5,065) | |  |  |
| --- | --- | --- | --- | --- | --- | --- |
| **Cancer Type** | N | Mean (SD) | N | Mean (SD) | **P**^1^ | **P**^2^ |
| **Oral Cavity and Pharynx** | 87 | **47.9 (7.80)** | 205 | **50.6 (9.48)** | **0.01013** | **0.01102** |
| **Digestive System** | 380 | 51.6 (8.99) | 822 | 50.8 (8.76) | 0.16324 | 0.08374 |
| Colon and Rectum | 169 | 50.8 (8.94) | 300 | 50.0 (8.68) | 0.35395 | 0.19682 |
| Pancreas | 74 | 51.9 (9.20) | 192 | 51.7 (8.61) | 0.86465 | 0.75366 |
| Other^a^ | 137 | 52.4 (8.92) | 330 | 51.0 (8.87) | 0.13583 | 0.11599 |
| **Respiratory System** | 202 | 51.9 (9.20) | 382 | 52.8 (9.03) | 0.28241 | 0.11686 |
| Lung and Bronchus | 174 | 52.3 (9.04) | 331 | 52.9 (9.01) | 0.53563 | 0.31333 |
| Other^b^ | 28 | 49.3 (9.92) | 51 | 52.2 (9.22) | 0.20939 | 0.40577 |
| **Skin^c^** | 148 | 47.4 (8.24) | 439 | 47.4 (8.79) | 0.99139 | 0.79915 |
| Melanoma of the skin | 128 | 47.4 (8.43) | 353 | 47.6 (8.58) | 0.86686 | 0.65793 |
| Other^d^ | 20 | 47.3 (7.06) | 86 | 46.8 (9.61) | 0.78451 | 0.64657 |
| **Breast** | 349 | 50.3 (8.32) | 785 | 50.4 (8.22) | 0.92609 | 0.53168 |
| **Female Genital System** | 99 | 49.5 (8.25) | 299 | 50.8 (8.70) | 0.19628 | 0.11948 |
| Corpus and Uterus | 44 | **47.5 (7.71)** | 136 | **50.5 (8.58)** | **0.03450** | 0.06539 |
| Ovary | 33 | 49.7 (8.53) | 104 | 50.2 (8.24) | 0.77344 | 0.66085 |
| Other^e^ | 22 | 53.3 (7.82) | 59 | 52.6 (9.60) | 0.72693 | 0.56276 |
| **Male Genital System** | 364 | 46.9 (8.68) | 662 | 47.4 (8.08) | 0.30469 | 0.29536 |
| Prostate | 337 | 46.9 (8.45) | 585 | 47.3 (7.97) | 0.45880 | 0.44076 |
| Other^f^ | 27 | 47.0 (11.33) | 77 | 48.6 (8.81) | 0.49592 | 0.36969 |
| **Urinary System** | 121 | 49.1 (8.88) | 306 | 50.1 (8.85) | 0.30609 | 0.17900 |
| Bladder | 53 | 50.1 (8.94) | 148 | 50.2 (8.56) | 0.95284 | 0.85742 |
| Kidney and Renal Pelvis | 66 | 48.1 (8.80) | 150 | 49.6 (9.15) | 0.25091 | 0.08510 |
| Other^g^ | 2 | 54.6 (9.02) | 8 | 56.1 (6.57) | 0.85895 | - |
| **Brain-nervous system** | 61 | 52.6 (7.41) | 177 | 52.1 (9.23) | 0.67104 | 0.64748 |
| **Endocrine System** | 54 | 50.0 (8.93) | 121 | 49.9 (8.54) | 0.95541 | 0.45616 |
| Thyroid | 48 | 49.5 (9.30) | 104 | 49.8 (8.67) | 0.87254 | 0.77554 |
| Other^h^ | 6 | 53.6 (3.94) | 17 | 50.6 (7.91) | 0.24775 | 0.93132 |
| **Lymphoma** | 78 | 50 (7.58) | 198 | 50.2 (8.42) | 0.80872 | 0.76312 |
| **Myeloma** | 54 | 50.8 (8.47) | 145 | 49.5 (8.8) | 0.35506 | 0.18171 |
| **Leukemia** | 77 | **47.2 (9.4)** | 234 | **49.7 (9.01)** | **0.03684** | **0.02438** |
| **Miscellaneous^i^** | 88 | 50.8 (9.43) | 190 | 50.8 (8.16) | 0.95762 | 0.92197 |

^1^Unadjusted t-test p-value; ^2^Adjusted for sex, age of cancer diagnosis, marital status, smoking status, cancer stage, BMI, and race/ethnicity; ^a^Esophagus, Stomach, Small Intestine, Liver, Intra Bile Duct, Anus, Gallbladder Other Biliary, Retroperitoneum, Peritoneum, Other Digestive Organs; ^b^Nose, Larynx, Pleura, Trachea, Other; ^c^excluding Basal and Squamous; ^d^Other Non-Epithelial, Squamous Cell Carcinoma; ^e^Cervix Uteri, Vagina, Vulva, Other Genital Organs; ^f^Testis, Penis, Other Genital Organs; ^g^Ureter, Other Urinary Organs; ^h^Other Endocrine System Organs; ^i^Kaposi Sarcoma, Mesothelioma, Eye Orbit, Soft tissue, Bone Joints, other Miscellaneous

**eTable 5:** Fatigue by cancer type and rural vs urban (n=7,271)

|  | **Rural** (n=2,206) | | **Urban** (n=5,065) | |  |  |
| --- | --- | --- | --- | --- | --- | --- |
| **Cancer Type** | N | Mean (SD) | N | Mean (SD) | **P**^1^ | **P**^2^ |
| **Oral Cavity and Pharynx** | 88 | 51.6 (11.00) | 207 | 54.8 (10.05) | **0.02358** | **0.01690** |
| **Digestive System** | 383 | 57.6 (10.09) | 825 | 56.7 (10.38) | 0.17305 | 0.12561 |
| Colon and Rectum | 171 | **56.8 (9.98)** | 300 | **54.7 (10.62)** | **0.03660** | **0.02379** |
| Pancreas | 74 | 58.5 (10.48) | 193 | 59.2 (9.39) | 0.65316 | 0.92973 |
| Other^a^ | 138 | 58.0 (10.01) | 332 | 57.1 (10.40) | 0.35431 | 0.39605 |
| **Respiratory System** | 200 | 58.5 (8.85) | 384 | 58.1 (9.51) | 0.66051 | 0.87866 |
| Lung and Bronchus | 172 | 59.1 (8.66) | 333 | 58.2 (9.35) | 0.30194 | 0.34368 |
| Other^b^ | 28 | 54.6 (9.15) | 51 | 57.4 (10.50) | 0.22554 | 0.18036 |
| **Skin^c^** | 150 | 48.8 (11.05) | 439 | 49.4 (10.61) | 0.54265 | 0.30345 |
| Melanoma of the skin | 130 | 48.9 (11.39) | 355 | 49.7 (10.58) | 0.49891 | 0.25154 |
| Other^d^ | 20 | 48.2 (8.80) | 84 | 48.5 (10.76) | 0.92717 | 0.77314 |
| **Breast** | 350 | 52.7 (9.57) | 790 | 53.1 (9.94) | 0.57628 | 0.37358 |
| **Female Genital System** | 101 | 54.0 (10.47) | 301 | 55.6 (9.75) | 0.18172 | 0.08898 |
| Corpus and Uterus | 45 | **51.6 (9.46)** | 138 | **54.7 (9.77)** | **0.05565** | 0.15002 |
| Ovary | 34 | 57.3 (9.37) | 104 | 56.8 (8.52) | 0.75839 | 0.68900 |
| Other^e^ | 22 | 54.1 (12.91) | 59 | 55.7 (11.54) | 0.59845 | 0.34277 |
| **Male Genital System** | 365 | 48.0 (10.41) | 665 | 49.1 (9.96) | 0.11156 | 0.14560 |
| Prostate | 340 | 48.0 (10.33) | 587 | 48.8 (9.81) | 0.21493 | 0.34870 |
| Other^f^ | 25 | 49.0 (11.67) | 78 | 51.2 (10.85) | 0.40957 | 0.31852 |
| **Urinary System** | 122 | 54.0 (10.85) | 304 | 54.7 (9.55) | 0.57749 | 0.16482 |
| Bladder | 53 | 53.5 (10.72) | 149 | 54.4 (8.25) | 0.56545 | 0.31369 |
| Kidney and Renal Pelvis | 67 | 54.2 (11.04) | 147 | 54.4 (10.52) | 0.91218 | 0.38929 |
| Other^g^ | 2 | 64.2 (0.35) | 8 | 65.2 (8.66) | 0.76890 | - |
| **Brain-nervous system** | 63 | 57.3 (9.26) | 177 | 57.6 (9.18) | 0.86771 | 0.83581 |
| **Endocrine System** | 53 | 51.4 (9.47) | 122 | 53.5 (10.60) | 0.19309 | 0.50988 |
| Thyroid | 47 | 51.6 (9.09) | 105 | 53.4 (10.77) | 0.30065 | 0.48008 |
| Other^h^ | 6 | 50.0 (13.05) | 17 | 54.6 (9.75) | 0.45340 | 0.12832 |
| **Lymphoma** | 79 | 56.5 (9.83) | 200 | 55.5 (9.38) | 0.45031 | 0.97422 |
| **Myeloma** | 54 | 57.5 (9.94) | 145 | 57.7 (8.69) | 0.85274 | 0.99247 |
| **Leukemia** | 77 | 53.2 (10.01) | 238 | 55.7 (10.08) | **0.05563** | **0.02665** |
| **Miscellaneous^i^** | 89 | 55.5 (10.87) | 190 | 54.9 (10.66) | 0.71842 | 0.70194 |

^1^Unadjusted t-test p-value; ^2^Adjusted for sex, age of cancer diagnosis, marital status, smoking status, cancer stage, BMI, and race/ethnicity; ^a^Esophagus, Stomach, Small Intestine, Liver, Intra Bile Duct, Anus, Gallbladder Other Biliary, Retroperitoneum, Peritoneum, Other Digestive Organs; ^b^Nose, Larynx, Pleura, Trachea, Other; ^c^excluding Basal and Squamous; ^d^Other Non-Epithelial, Squamous Cell Carcinoma; ^e^Cervix Uteri, Vagina, Vulva, Other Genital Organs; ^f^Testis, Penis, Other Genital Organs; ^g^Ureter, Other Urinary Organs; ^h^Other Endocrine System Organs; ^i^Kaposi Sarcoma, Mesothelioma, Eye Orbit, Soft tissue, Bone Joints, other Miscellaneous

**eTable 6:** Pain Inference by cancer type and rural vs urban (n=7,271)

|  | **Rural** (n=2,206) | | **Urban** (n=5,065) | |  |  |
| --- | --- | --- | --- | --- | --- | --- |
| **Cancer Type** | N | Mean (SD) | N | Mean (SD) | **P**^1^ | **P**^2^ |
| **Oral Cavity and Pharynx** | **88** | **52.8 (10.55)** | **209** | **55.4 (9.15)** | **0.04567** | **0.02064** |
| **Digestive System** | 384 | 56.5 (10.17) | 831 | 55.5 (10.07) | 0.10808 | 0.10975 |
| Colon and Rectum | **171** | **56.0 (10.27)** | **301** | **53.7 (10.19)** | **0.01818** | **0.02172** |
| Pancreas | 75 | 56.8 (10.63) | 193 | 58.6 (9.38) | 0.20248 | 0.51995 |
| Other^a^ | 138 | 57.0 (9.85) | 337 | 55.4 (9.95) | 0.10714 | 0.19212 |
| **Respiratory System** | 200 | 57.0 (9.60) | 386 | 57.0 (9.53) | 0.97912 | 0.85374 |
| Lung and Bronchus | 172 | 57.5 (9.68) | 334 | 56.8 (9.70) | 0.50026 | 0.52918 |
| Other^b^ | **28** | **53.8 (8.61)** | **52** | **57.8 (8.35)** | **0.05242** | **0.02491** |
| **Skin^c^** | 150 | 50.3 (9.18) | 443 | 50.0 (9.93) | 0.70485 | 0.63217 |
| Melanoma of the skin | 130 | 50.4 (9.13) | 357 | 50.4 (9.98) | 0.99976 | 0.30205 |
| Other^d^ | 20 | 49.6 (9.75) | 86 | 48.1 (9.53) | 0.54934 | 0.30849 |
| **Breast** | 349 | 52.0 (9.14) | 793 | 52.6 (8.99) | 0.29916 | 0.17516 |
| **Female Genital System** | 101 | 55.0 (10.31) | 302 | 54.5 (9.41) | 0.66410 | 0.77013 |
| Corpus and Uterus | 45 | 53.5 (9.07) | 139 | 54.1 (9.99) | 0.74680 | 0.88335 |
| Ovary | 34 | 55.3 (10.88) | 104 | 54.9 (8.64) | 0.83671 | 0.82051 |
| Other^e^ | 22 | 57.7 (11.64) | 59 | 55.0 (9.42) | 0.34657 | 0.57784 |
| **Male Genital System** | 366 | 49.2 (8.91) | 666 | 50.1 (9.17) | 0.10250 | 0.10882 |
| Prostate | 341 | 49.1 (8.86) | 588 | 49.9 (9.08) | 0.17349 | 0.25875 |
| Other^f^ | 25 | 49.8 (9.76) | 78 | 51.4 (9.75) | 0.46403 | 0.24208 |
| **Urinary System** | 122 | 55.2 (9.77) | 305 | 54.5 (9.36) | 0.48550 | 0.74204 |
| Bladder | 53 | 54.1 (9.34) | 149 | 54.3 (9.36) | 0.92725 | 0.96935 |
| Kidney and Renal Pelvis | 67 | 55.8 (10.08) | 148 | 54.2 (9.22) | 0.26776 | 0.63729 |
| Other^g^ | 2 | 65.0 (4.24) | 8 | 64.8 (6.97) | 0.95323 | - |
| **Brain-nervous system** | 63 | 53.0 (9.09) | 177 | 53.6 (9.90) | 0.63820 | 0.58921 |
| **Endocrine System** | 53 | 50.0 (8.69) | 124 | 50.4 (10.09) | 0.77537 | 0.88940 |
| Thyroid | 47 | 49.2 (8.05) | 107 | 50.1 (10.35) | 0.58384 | 0.85468 |
| Other^h^ | 6 | 56.0 (11.87) | 17 | 52.6 (8.12) | 0.53853 | 0.92361 |
| **Lymphoma** | 79 | 53.3 (10.06) | 201 | 53.8 (9.74) | 0.70438 | 0.31845 |
| **Myeloma** | 54 | 58.9 (9.14) | 146 | 57.8 (7.9) | 0.44313 | 0.45054 |
| **Leukemia** | 79 | 50.7 (9.64) | 238 | 52.5 (9.45) | 0.14514 | 0.09704 |
| **Miscellaneous^i^** | 89 | 57.2 (9.82) | 191 | 55.8 (9.54) | 0.28463 | 0.37187 |

^1^Unadjusted t-test p-value; ^2^Adjusted for sex, age of cancer diagnosis, marital status, smoking status, cancer stage, BMI, and race/ethnicity; ^a^Esophagus, Stomach, Small Intestine, Liver, Intra Bile Duct, Anus, Gallbladder Other Biliary, Retroperitoneum, Peritoneum, Other Digestive Organs; ^b^Nose, Larynx, Pleura, Trachea, Other; ^c^excluding Basal and Squamous; ^d^Other Non-Epithelial, Squamous Cell Carcinoma; ^e^Cervix Uteri, Vagina, Vulva, Other Genital Organs; ^f^Testis, Penis, Other Genital Organs; ^g^Ureter, Other Urinary Organs; ^h^Other Endocrine System Organs; ^i^Kaposi Sarcoma, Mesothelioma, Eye Orbit, Soft tissue, Bone Joints, other Miscellaneous

**eTable 7:** Physical Function by cancer type and rural vs urban (n=7,271)

|  | **Rural** (n=2,206) | | **Urban** (n=5,065) | |  |  |
| --- | --- | --- | --- | --- | --- | --- |
| **Cancer Type** | N | Mean (SD) | N | Mean (SD) | **P**^1^ | **P**^2^ |
| **Oral Cavity and Pharynx** | **89** | **48.0 (9.98)** | **209** | **44.6 (10.33)** | **0.00891** | **0.00269** |
| **Digestive System** | 386 | 41.7 (9.68) | 834 | 42.2 (9.82) | 0.34643 | 0.48064 |
| Colon and Rectum | **172** | **42.2 (9.21)** | **303** | **44.4 (9.98)** | **0.01282** | **0.04460** |
| Pancreas | 75 | 40.4 (9.07) | 193 | 39.4 (8.76) | 0.42076 | 0.78111 |
| Other^a^ | 139 | 41.7 (10.53) | 338 | 41.9 (9.81) | 0.89625 | 0.98079 |
| **Respiratory System** | 200 | 38.2 (8.23) | 387 | 39.5 (9.18) | 0.09229 | 0.08146 |
| Lung and Bronchus | **172** | **37.6 (8.05)** | **335** | **39.3 (9.22)** | **0.02868** | **0.01451** |
| Other^b^ | 28 | 42.5 (8.20) | 52 | 40.9 (8.94) | 0.40847 | 0.23190 |
| **Skin^c^** | 150 | 50.5 (10.49) | 442 | 50.8 (11.08) | 0.79505 | 0.73843 |
| Melanoma of the skin | 130 | 50.5 (10.65) | 358 | 50.6 (10.91) | 0.94013 | 0.58931 |
| Other^d^ | 20 | 50.7 (9.60) | 84 | 51.6 (11.83) | 0.69943 | 0.83198 |
| **Breast** | 350 | 47.8 (10.03) | 796 | 47.0 (9.88) | 0.24449 | 0.04891 |
| **Female Genital System** | 101 | 43.2 (10.59) | 307 | 43.2 (9.60) | 0.97018 | 0.67861 |
| Corpus and Uterus | 45 | 43.2 (9.70) | 142 | 43.6 (9.83) | 0.84761 | 0.86305 |
| Ovary | 34 | 41.4 (9.72) | 106 | 41.4 (8.63) | 0.98521 | 0.54267 |
| Other^e^ | 22 | 45.8 (13.28) | 59 | 45.7 (10.23) | 0.98414 | 0.74916 |
| **Male Genital System** | **368** | **51.9 (9.28)** | **668** | **50.5 (10.03)** | **0.02080** | **0.01692** |
| Prostate | **343** | **51.9 (9.28)** | 590 | **50.6 (9.96)** | **0.03870** | 0.08272 |
| Other^f^ | 25 | 51.7 (9.51) | 78 | 49.7 (10.58) | 0.36141 | 0.03895 |
| **Urinary System** | 123 | 43.6 (10.57) | 305 | 44.2 (9.34) | 0.62527 | 0.89178 |
| Bladder | 54 | 45.1 (10.05) | 149 | 44.6 (9.69) | 0.72595 | 0.30577 |
| Kidney and Renal Pelvis | 67 | 42.6 (11.02) | 148 | 44.4 (8.67) | 0.24264 | 0.19051 |
| Other^g^ | 2 | 38.0 (2.83) | 8 | 32.5 (7.93) | 0.16706 | - |
| **Brain-nervous system** | 63 | 41.2 (9.88) | 178 | 40.7 (10.68) | 0.72910 | 0.36643 |
| **Endocrine System** | 54 | 50.5 (9.56) | 124 | 50.1 (10.43) | 0.81838 | 0.86365 |
| Thyroid | 48 | 51.5 (8.71) | 107 | 50.7 (10.04) | 0.61225 | 0.55169 |
| Other^h^ | 6 | 42.7 (13.20) | 17 | 46.8 (12.43) | 0.52995 | 0.52806 |
| **Lymphoma** | 80 | 45.1 (10.48) | 202 | 44.4 (9.69) | 0.64651 | 0.16099 |
| **Myeloma** | 54 | - | 146 | - | - | 0.28835 |
| **Leukemia** | 79 | 42.9 (10.78) | 241 | 42.2 (9.55) | 0.49940 | 0.05820 |
| **Miscellaneous^i^** | 90 | 42.5 (11.42) | 191 | 42.0 (10.02) | 0.75412 | 0.79963 |

^1^Unadjusted t-test p-value; ^2^Adjusted for sex, age of cancer diagnosis, marital status, smoking status, cancer stage, BMI, and race/ethnicity; ^a^Esophagus, Stomach, Small Intestine, Liver, Intra Bile Duct, Anus, Gallbladder Other Biliary, Retroperitoneum, Peritoneum, Other Digestive Organs; ^b^Nose, Larynx, Pleura, Trachea, Other; ^c^excluding Basal and Squamous; ^d^Other Non-Epithelial, Squamous Cell Carcinoma; ^e^Cervix Uteri, Vagina, Vulva, Other Genital Organs; ^f^Testis, Penis, Other Genital Organs; ^g^Ureter, Other Urinary Organs; ^h^Other Endocrine System Organs; ^i^Kaposi Sarcoma, Mesothelioma, Eye Orbit, Soft tissue, Bone Joints, other Miscellaneous

**eTable 8:** PRO scores by gender and rurality status

| **PROMIS** | **Women** (n=3,505) | | | | | | **Men** (n=3,765) | | | | | |
| --- | --- | --- | --- | --- | --- | --- | --- | --- | --- | --- | --- | --- |
|  | Rural | | Urban | |  |  | Rural | | Urban | |  |  |
|  | N | Mean (SD) | N | Mean (SD) | β ± SE^§^ | *P*^§^ | N | Mean (SD) | N | Mean (SD) | β ± SE | *P* |
| **Anxiety** | 990 | 55.3 (8.82) | 2,453 | 55.5 (8.98) | -0.435 ± 0.34 | 0.2038 | 1,181 | 52.6 (9.09) | 2,520 | 52.5 (9.09) | 0.07 ± 0.32 | 0.8264 |
| **Depression** | 987 | 50.9 (8.43) | 2,449 | 51.0 (8.50) | -0.396 ± 0.32 | 0.2215 | 1,175 | 48.7 (9.02) | 2,515 | 49.0 (8.91) | -0.339 ± 0.32 | 0.2857 |
| **Fatigue** | 989 | 55.0 (10.2) | 2,460 | 55.2 (10.2) | -0.433 ± 0.38 | 0.2512 | 1,185 | 52.5 (11.0) | 2,526 | 53.1 (10.6) | -0.482 ± 0.36 | 0.1761 |
| **Pain interference** | 989 | 54.0 (9.93) | 2,473 | 53.9 (9.73) | -0.123 ± 0.37 | 0.7378 | 1,188 | 52.9 (10.0) | 2,538 | 53.1 (9.79) | -0.231 ± 0.34 | 0.4940 |
| **Physical function** | 994 | 44.3 (10.6) | 2,485 | 44.1 (10.3) | 0.56 ± 0.36 | 0.1177 | 1,193 | 46.5 (10.9) | 2,544 | 46.1 (10.8) | 0.41 ± 0.34 | 0.2298 |

**eTable 9:** PRO scores by race/ethnicity and rurality status

| **PROMIS** | **Non-Hisp White** (n=6,403) | | | | | | **Other**^§^ (n=718) | | | | | |
| --- | --- | --- | --- | --- | --- | --- | --- | --- | --- | --- | --- | --- |
|  | Rural | | Urban | |  |  | Rural | | Urban | |  |  |
|  | N | Mean (SD) | N | Mean (SD) | β ± SE^$^ | *P* | N | Mean (SD) | N | Mean (SD) | β ± SE^&^ | *P* |
| **Anxiety** | 1,981 | 53.7 (8.99) | 4,325 | 54.0 (9.09) | -0.278 ± 0.24 | 0.2520 | 146 | 55.7 (9.85) | 545 | 54.4 (9.50) | 1.402 ± 0.89 | 0.1176 |
| **Depression** | 1,972 | 49.5 (8.67) | 4,316 | 50.0 (8.69) | -0.48 ± 0.23 | **0.0405** | 146 | 51.2 (10.2) | 545 | 50.2 (9.16) | 1.028 ± 0.88 | 0.2437 |
| **Fatigue** | 1,982 | 53.5 (10.7) | 4,336 | 54.3 (10.4) | -0.732 ± 0.27 | **0.0065** | 147 | 55.1 (10.9) | 547 | 52.9 (10.6) | 1.671 ± 0.98 | 0.0878 |
| **Pain interference** | 1,986 | 53.2 (9.92) | 4,354 | 53.3 (9.73) | -0.256 ± 0.26 | 0.3226 | 146 | 55.9 (10.2) | 553 | 54.8 (9.82) | 0.687 ± 0.91 | 0.4502 |
| **Physical function** | 1,994 | 45.8 (10.8) | 4,366 | 45.2 (10.6) | 0.662 ± 0.26 | **0.0099** | 148 | 43.0 (10.8) | 559 | 44.7 (10.5) | -1.506 ± 0.91 | 0.0990 |

**eTable 10:** PRO scores by marital status and rurality status

| **PROMIS** | **Other^ꝉ^** (n=2,006) | | | | | | **Married^ꝉ^** (n=5,085) | | | | | |
| --- | --- | --- | --- | --- | --- | --- | --- | --- | --- | --- | --- | --- |
|  | Rural | | Urban | |  |  | Rural | | Urban | |  |  |
|  | N | Mean (SD) | N | Mean (SD) | β ± SE^$^ | *P* | N | Mean (SD) | N | Mean (SD) | β ± SE^&^ | *P* |
| **Anxiety** | 555 | 55.5 (9.40) | 1,414 | 55.2 (9.27) | 0.293 ± 0.46 | 0.5277 | 1,565 | 52.6 (9.09) | 3433 | 52.5 (9.09) | -0.333 ± 0.27 | 0.2187 |
| **Depression** | 552 | 51.6 (9.27) | 1,410 | 51.1 (8.95) | 0.303 ± 0.45 | 0.5043 | 1,559 | 48.7 (9.02) | 3427 | 49.0 (8.91) | -0.647 ± 0.26 | **0.0132** |
| **Fatigue** | 559 | 55.9 (10.7) | 1,416 | 55.6 (10.4) | 0.31 ± 0.50 | 0.5367 | 1,564 | 52.5 (11.0) | 3444 | 53.1 (10.6) | -0.79 ± 0.30 | **0.0091** |
| **Pain interference** | 558 | 55.7 (10.1) | 1,425 | 54.6 (9.98) | 1.11 ± 0.48 | **0.0211** | 1,567 | 52.9 (10.0) | 3460 | 53.1 (9.79) | -0.642 ± 0.29 | **0.0269** |
| **Physical function** | 562 | 43.1 (10.7) | 1,435 | 43.6 (10.4) | -0.444 ± 0.47 | 0.3417 | 1,573 | 46.5 (10.9) | 3468 | 46.1 (10.8) | 0.857 ± 0.29 | **0.0032** |

^§^Adjusted for age at cancer diagnosis, sex, BMI, marital status, smoking status, cancer stage at diagnosis, and cancer type
